# Identification of new enterosynes using prebiotics: roles of bioactive lipids and mu-opioid receptor signalling in humans and mice

**DOI:** 10.1136/gutjnl-2019-320230

**Published:** 2020-10-05

**Authors:** Anne Abot, Eve Wemelle, Claire Laurens, Adrien Paquot, Nicolas Pomie, Deborah Carper, Arnaud Bessac, Xavier Mas Orea, Christophe Fremez, Maxime Fontanie, Alexandre Lucas, Jean Lesage, Amandine Everard, Etienne Meunier, Gilles Dietrich, Giulio G Muccioli, Cedric Moro, Patrice D Cani, Claude Knauf

**Affiliations:** 1 IRSD, INSERM, Toulouse, Occitanie, France; 2 Enterosys, CRO, Toulouse, Occitanie, France; 3 European Associated Laboratory (EAL) NeuroMicrobiota, Toulouse, Brussels, France, Belgium; 4 CNRS, University of Strasbourg, Strasbourg, France; 5 CNES, Paris, France; 6 Bioanalysis and Pharmacology of Bioactive Lipids Research Group, Louvain Drug Research Institute, UCLouvain, Université catholique de Louvain, Brussels, Belgium; 7 I2MC, INSERM, Toulouse, Occitanie, France; 8 IPBS, Toulouse, Midi-Pyrénées, France; 9 Lille 2 University of Health and Law, Lille, Hauts-de-France, France; 10 Metabolism and Nutrition Research Group, Louvain Drug Research Institute, Walloon Excellence in Life Sciences and BIOtechnology (WELBIO), UCLouvain, Université catholique de Louvain, Brussels, Belgium

**Keywords:** enteric nervous system, diabetes mellitus, prebiotic, lipids, motility disorders

## Abstract

**Objective:**

The enteric nervous system (ENS) plays a key role in controlling the gut-brain axis under normal and pathological conditions, such as type 2 diabetes. The discovery of intestinal actors, such as enterosynes, able to modulate the ENS-induced duodenal contraction is considered an innovative approach. Among all the intestinal factors, the understanding of the role of gut microbes in controlling glycaemia is still developed. We studied whether the modulation of gut microbiota by prebiotics could permit the identification of novel enterosynes.

**Design:**

We measured the effects of prebiotics on the production of bioactive lipids in the intestine and tested the identified lipid on ENS-induced contraction and glucose metabolism. Then, we studied the signalling pathways involved and compared the results obtained in mice to human.

**Results:**

We found that modulating the gut microbiota with prebiotics modifies the actions of enteric neurons, thereby controlling duodenal contraction and subsequently attenuating hyperglycaemia in diabetic mice. We discovered that the signalling pathway involved in these effects depends on the synthesis of a bioactive lipid 12-hydroxyeicosatetraenoic acid (12-HETE) and the presence of mu-opioid receptors (MOR) on enteric neurons. Using pharmacological approaches, we demonstrated the key role of the MOR receptors and proliferator-activated receptor γ for the effects of 12-HETE. These findings are supported by human data showing a decreased expression of the proenkephalin and MOR messanger RNAs in the duodenum of patients with diabetic.

**Conclusions:**

Using a prebiotic approach, we identified enkephalin and 12-HETE as new enterosynes with potential real beneficial and safety impact in diabetic human.

**Significance of this study**

**What is already known on this subject?**

- Targeting the ‘enteric nervous system (ENS)/duodenal contraction’ couple is considered as an innovative therapeutic strategy to treat type 2 diabetes.
- ENS is under the influence of various molecules from the gut also called enterosynes, which have the capacity to decrease duodenal contraction.
- Gut microbiota is well known to have an antidiabetic action through hormonal, nervous and metabolic regulations.

**What are the new findings?**

- Modulation of gut microbiota composition by prebiotics improves glucose homeostasis by acting on ENS/duodenal contraction couple.
- The mechanism of prebiotic action implies the presence of an enkephalin/mu-opioid receptor and proliferator-activated receptor γ signalling and a bioactive lipid, 12S-hydroxyeicosatetraenoic acid.
- The expression of enzymes implicated in enteric neurotransmitter synthesis and enkephalin signalling are altered in the duodenum of diabetic human.

**How might it impact on clinical practice in the foreseeable future?**

- This study is the first showing the alteration of ENS and enkephalin signalling in the duodenum of diabetic human. Deciphering the mode of action of gut microbiota on ENS could represent a real safety and innovative therapeutic evidence for patients with diabetic.

## Introduction

The identification of new targets to treat type 2 diabetes (T2D) is considered of major importance for public health. Although numerous bioactive pharmacological molecules have been approved and used as antidiabetics, the large majority of these molecules has side effects.^1 2^ In addition to the limitation of deleterious effects, future therapeutic strategies should preferentially be administered to patients via the oral route.^3^

Recently, a new concept has emerged: the enteric nervous system (ENS) is considered as new target to treat T2D.^4^ In fact, duodenal hypercontractility observed during T2D leads to the genesis of aberrant signalling from the afferent nerves to the hypothalamus, contributing to systemic insulin resistance.^5^ Using pharmacological approaches, an oral treatment with gut peptides has been shown to improve glucose metabolism by stimulating the release of nitric oxide (NO) from enteric neurons. Thus, enteric NO has the capacity to decrease duodenal contractions and restore the gut-brain axis, subsequently improving insulin sensitivity.^5 6^ Therefore, the identification of intestinal bioactive molecules that are able to target the ENS, also called enterosynes,^7^ represents an innovative therapeutic approach.

Since the beginning of the 2000s, accumulating evidence has revealed key roles for the gut microbiota and its metabolites in controlling glucose metabolism.^8 9^ Currently, the identification of one bacteria and/or one of its active metabolites is viewed as potential novel therapeutic strategy.^10^ Using nutritional approaches, gut microbiota remodelling with prebiotics and/or probiotics improves glucose metabolism in subjects with T2D.^8 11^ A potential explanation for this change is the release of various factors (bioactive peptides/lipids, neurotransmitters, gases and hormones) from bacteria and from the host that are able to decrease hepatic steatosis and adipose tissue inflammation, among other effects.^9 12^

The activity of enteric neurons is modulated (1) directly by the bacteria, which are sensed by intrinsic primary afferent neurons^13^ or (2) through the release of bioactive molecules whose main representatives are short chain fatty acids (SCFAs).^8^ The discovery of new enterosynes that are able to improve glucose metabolism by modulating the activity of ENS neurons has been highlighted as a promising future source of antidiabetic drugs.^4 7 14^ Actually, researchers have not determined the real potential of strategies modulating the gut microbiota using prebiotics to provide novel enterosynes. Therefore, in the present study, we have used a prebiotic treatment (ie, oligofructose) to identify novel actors and the molecular mechanisms explaining their antidiabetic properties. We found that prebiotic treatment decreases duodenal hypercontractility by modulating ENS activity. Using lipidomic approaches, we discovered that the improvement in the diabetic state was associated with an increase in the levels of an intestinal lipid, 12-hydroxyeicosatetraenoic acid (12-HETE). We then tested the impact of chronic oral administration of 12-HETE on gut hypercontractility and the effects on the duodenal expression of neuronal NO synthase (nNOS). In addition, given that 12-HETE is considered a second messenger that transmits signals from activated mu-opioid receptors (MOR) and subsequently increases the activity of potassium channels to inhibit neurotransmitter release in neurons,^15^ we used pharmacological approaches to modulate both MOR and also the putative nuclear receptor targeted by 12-HETE, that is, peroxisome proliferator-activated receptor γ (PPARγ). Finally, we investigated whether the expression of the proenkephalin (PENK) messanger RNA (mRNA) encoding the one of the two endogenous ligands of MORthat is, enkephalin, in the duodenum of patients with diabetes.

## Materials and methods

### Human duodenum

The postmortem duodenal biopsies were obtained from Caucasian men aged between 42 and 67 years at times ranging from 6 to 12 hour after death (Tebu-Bio, Le Perray-en-Yvelines, France) in accordance with the regulations of the French Ministry (Authorization AC-2018-3108) and with the consent of the individuals (Tebu-Bio). The donors were patients with diabetes who received antidiabetic treatments or healthy volunteers who never received antidiabetic treatments.

### Mice

**Figure 1:**
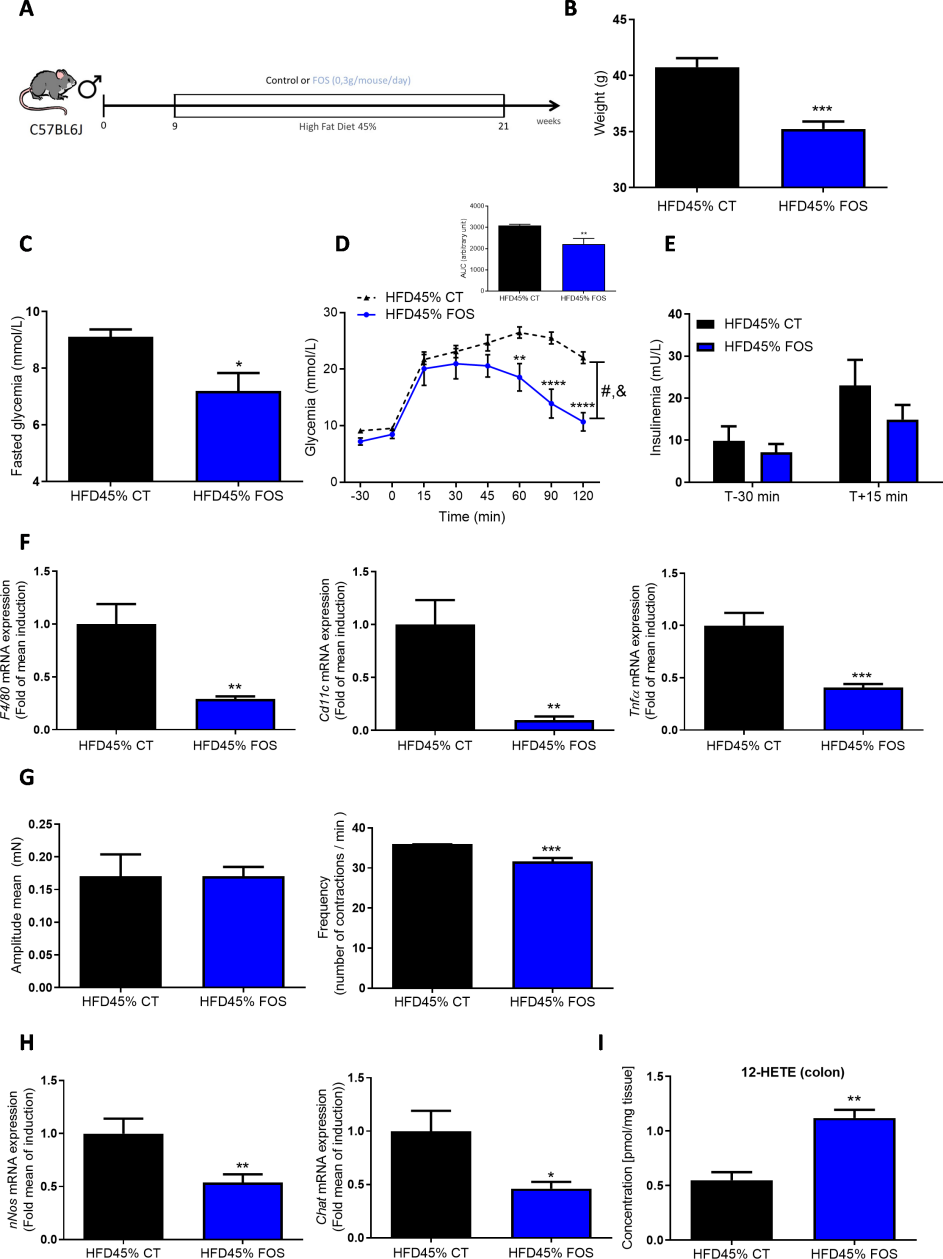
FOS improves glucose metabolism and adipose tissue inflammation. (A) Experiment designed to explore the metabolic effects of FOS in diabetic mice fed a HFD45%. (B) Body weight at the end of the experimental protocol (n=18–19 animals per group). (C) The results for fasting glycaemia. (D) The oral glucose tolerance test (OGTT) in mice that had fasted for 6 hour along with the area under the curve. (E) Plasma insulin levels in mice treated with or without FOS are shown (n=5–7 per group). (F) Expression of the *F4/80*, *Cd11c* and *Tnfα* mRNAs in the eWAT (n=7–8 animals per group). (G) Ex vivo measurement of the duodenal mechanical contraction amplitude and frequency (n=5–7 samples per group). (H) Expression of the *nNos* and *Chat* mRNAs in the duodenum (n=13 samples per group). (I) Concentration of 12-HETE in the colon (n=5 samples per group). *p<0.05, **p<0.01, ***p<0.001 and ****p<0.0001 compared with HFD45% CT; time effect: ^&^p<0.001 and treatment effect ^#^p<0.001 (two-way ANOVA). ANOVA, analysis of variance; FOS, fructooligosaccharides; HFD, high-fat diet; 12-HETE, 12-hydroxyeicosatetraenoicacid.

Nine-week-old male C57BL/6J mice (Charles River Laboratory, l’Arbresle, France) were housed under specific pathogen-free conditions in controlled environment (room temperature of 23°C±2°C, 12 hours daylight cycle) and were provided free access to food and water. Experiments were conducted according to the European Community Regulations concerning the protection of experimental animals and were approved by the local Animal Care and Use Committee. Mice were fed a high-fat diet (HFD) containing 20% protein, 35% carbohydrate and 45% fat (Research Diet, New Brunswick, New Jersey, USA) supplemented with or without prebiotics (fructooligosaccharides (FOS), ORAFTI P95, 0.3 g per mouse per day added to the tap water, Orafti, Tienen, Belgium) as described in our previous study^16^ (figure 1A). Oral gavage of 12S-HETE (100 µL, final concentration of 1 µM, Cayman Chemical, Michigan, USA) or DAMGO ((D-Ala2, NMe-Phe4, Gly-ol5)-enkephalin, a highly selective MOR agonist, 100 µL, final concentration of 100 nM, Sigma-Aldrich, Michigan, USA) was performed daily in HFD45%-treated mice during the last week of the HFD45% treatment. The same timing was used for intraperitoneal injection of GW9662 (1 mg/kg/day during the last week). All protocols described below were performed at the end of the HFD45% treatment.

### Measurement of isotonic contractions

Mice were euthanised under fed conditions. After dissection, the duodenum was washed and incubated with an oxygenated Krebs-Ringer solution, pH 7.4, for 30 min at 37°C, attached to the isotonic transducer (MLT7006 Isotonic Transducer, Hugo Basile, Comerio, Italy), and immersed in an organ bath containing the same medium maintained at 37°C. The load applied to the lever was 1 g (10 mN). Isotonic contractions were recorded using Labchart software (AD Instruments) following transducer displacement. After attaching the intestinal segments, basal contractions were recorded for 10 min. For acute treatments, 100 µL of Krebs-Ringer (vehicle) solution or specific drugs (12-HETE or DAMGO) were added to the survival medium, and contractions were recorded for 10 min. Contraction amplitudes are presented as percentages relative to the basal response (before the injection of vehicle or drugs), while contraction frequencies are presented as numbers of contractions per minute.

### Oral glucose tolerance test

Mice that had fasted for 6 hour were orally loaded with glucose (3 g/kg of body weight). Glycaemia was measured at −30, 0,+15,+30,+60 and +90 min with a glucometer (Accu-Chek Active, Roche), and blood was collected from the tail vein at −30 min and +15 min to measure plasma insulin levels. Insulin levels were analysed using homogeneous time resolved fluorescence (HTRF) serum kits (Cisbio, France), as previously described.^6^

### Tissue-specific (2-3H) deoxyglucose uptake in vivo

Tissue-specific glucose uptake was assessed in response to an intraperitoneal bolus injection of 2-[1,2-3H(N)]deoxy-D-glucose (PerkinElmer, Boston, Massachusetts, USA) (0.4 µCi/g body weight) and insulin (3 µ/g body weight). The dose of insulin was determined in preliminary studies to reach a nearly maximal stimulation of insulin signalling and glucose uptake in all muscle types and metabolic tissues. Mice were fasted for 2 hour before the injection and euthanised 30 min after the injection; tissues were extracted by precipitation using 2-deoxyglucose-6-phosphate, as previously described.^17^

### Lipid assay

Targeted lipidomics was performed in collaboration with Biocrates (Innsbruck, Austria). The different bioactive lipids (ie, 12-HETE, 6-keto-PGF_1a_, 8-iso-PGF_2a_, 9-HODE, 13-HODE, 15-HETE, AA, DHA, LTB_4_, PGD_2_, PGE_2_, PGF_2a_ and TXB_2_) were quantitatively analysed using a high-throughput flow injection electrospray ionisation mass spectrometry (ESI-MS/MS) screening method. Multiple reaction monitoring (MRM) detection in positive and negative modes was performed using an AB SCIEX 4000 QTrap tandem mass spectrometry instrument (AB SCIEX, Darmstadt, Germany). The sample was prepared in a 20 µL volume, followed by an MeOH/CHCl_3_-liquid/liquid-extraction protocol. Internal standards were used to compensate for matrix effects, and external standards were used for multipoint calibration. The quantitative analysis was performed with Biocrates in-house software MetIDQ enabling isotopic correction.

### Immunohistochemistry

Adult male C57BL/6 mice were euthanised by the administration of a lethal dose of anaesthesia and their duodenal tissues were fixed with a 4% formalin solution (Sigma-Aldrich) for 16 hours and maintained at 4°C in 70% ethanol until they were embedded in paraffin. Six-micron-thick sections were prepared. After a citrate pretreatment, sections were incubated with goat anti-nNOS (1/100, ab1376, Abcam), sheep anti-ChAT (Choline Acetyl Transferase, 1/100, ab18736, Abcam), rabbit anti-MOR (1/100, ab10275, Abcam) or rabbit anti-Alox12 (1/100, bs3874R, Bioss) primary antibodies for 16 hours at 4°C. After washes with 1X phosphate-buffered saline (PBS), sections were incubated with fluorescein isothiocyanate (FITC)-conjugated species-specific secondary antibodies (Jackson ImmunoResearch Laboratories, West Grove, Pennsylvania, USA) to detect MOR or Alox12 labelling and with tetramethylrhodamine (TRITC) secondary antibodies (Jackson Laboratories) to detect ChAT labelling in dual labelling experiments. For MOR and nNOS dual labelling and Alox12 and nNOS dual labelling, sections were incubated with FITC-conjugated secondary antibodies to detect nNOS labelling and with TRITC-conjugated secondary antibodies to detect MOR/Alox12 one. Nuclei (blue) were counterstained with Hoechst 33 258. High-quality fluorescence images were captured with a Leica DM5500B microscope using a 100× oil objective.

### Gene expression

Tissues were homogenised using a Precellys tissue homogeniser (Bertin Technol., Montigny-le-Bretonneux, France), and total RNA was extracted from tissues using TRIReagent (Sigma-Aldrich) and a GenElute Mammalian Total RNA Miniprep Kit (Sigma-Aldrich), according to the manufacturers’ instructions. The complementary DNAs (cDNAs) were generated either using an Moloney murine leukemia virus reverse transcriptase (M-MLV) Reverse transcriptase kit (Invitrogen) and random hexamers (Invitrogen) or High-capacity cDNA reverse transcription kit (ThermoFisher Scientific). Real-time PCR was performed with a LightCycler 480 (Life Technologies) or ViiA 7 Real-Time PCR System using SYBR Green Real-Time PCR Master Mixes (Thermo Fisher Scientific) or Taqman PCR Fast Advanced Master Mix and primers that were validated by testing the PCR efficiency. The sequences of primers used for cDNA amplification in the quantitative RT-PCR experiments are listed in online supplemental table 1. Assays on demand were used to detect F4/80 (Mm00802530_m1), TNFa (Mm00443258_m1) and Pdk4 (Mm00443325_m1) (ThermoFischer Scientific). Gene expression was quantified using the comparative Ct (threshold cycle) method. The results were normalised to beta-2-microglobulin expression. The identity and purity of the amplified product were assessed by analysing the melting curve, which was performed at the end of amplification.

10.1136/gutjnl-2019-320230.supp1 Supplementary data

### Statistical analysis

The data are presented as means±SEM. Differences between the experimental groups were assessed using unpaired Student’s t-tests and one-way or two-way analysis of variance, followed by post hoc tests, as appropriate. Data were analysed using GraphPad Prism software for Windows (GraphPad Software, San Diego, California, USA). The results were considered statistically significant at p<0.05.

## Results

### FOS improves glucose tolerance, reduces ENS activity and duodenal contraction

We first assessed the effect of prebiotic-induced modulation of the gut microbiota on ENS function. Here, we confirmed that a chronic treatment with FOS prebiotics in diabetic mice fed a HFD45% (figure 1A) leads to a decrease in body weight (figure 1B and online supplemental figure S1A) associated with a decrease in fasting hyperglycaemia (figure 1C). These changes were linked to an improvement in glucose tolerance (figure 1D), without a change in insulin release (figure 1E). A decrease in the mRNA expression of inflammatory markers, that is, *F4/80*, *Cd11c* and *Tnfα* (figure 1F), was also observed in the epididymal white adipose tissue (eWAT). In this tissue, no variation of mRNA expression of mitochondrial function (*Ucp1*, *Prdm16*, *Pdk4*, *Pgc1a*) was shown (online supplemental figure S2A). No significant difference was observed in the expression of metabolism-related mRNAs in the liver, muscles and brown adipose tissue (online supplemental figure S2B–D).

Interestingly, real-time measurements of duodenal contraction revealed that the FOS treatment reduced duodenal contraction frequency (figure 1G) and associated with a decrease in *nNos* and *Chat* mRNA expression (figure 1H). Based on these findings, although FOS prebiotics are fermented in the lower part of the gut, they are able to act at distance from the colon and therefore modify the neurochemical transmission of the gastrointestinal tract *via* putative pathways mediated by the endocrine and/or nervous systems.

Therefore, we decided to investigate which potential bioactive molecule(s) explained the effect of FOS on gut contraction. We used a lipidomic approach to measure the quantities of various bioactive lipids present in the colon of mice fed a HFD45% and supplemented with the prebiotic FOS. Among the different lipids measured (online supplemental figure S3), the prebiotic treatment selectively increased the levels of 12-HETE in HFD45%+FOS-fed mice (figure 1I), but not in the duodenum (online supplemental figure S1B).

We next investigated whether the increased 12-HETE concentration contributed to the observed phenotype by measuring duodenal contractility ex vivo.

### 12S-HETE controls duodenal contraction and improves glucose tolerance

**Figure 2:**
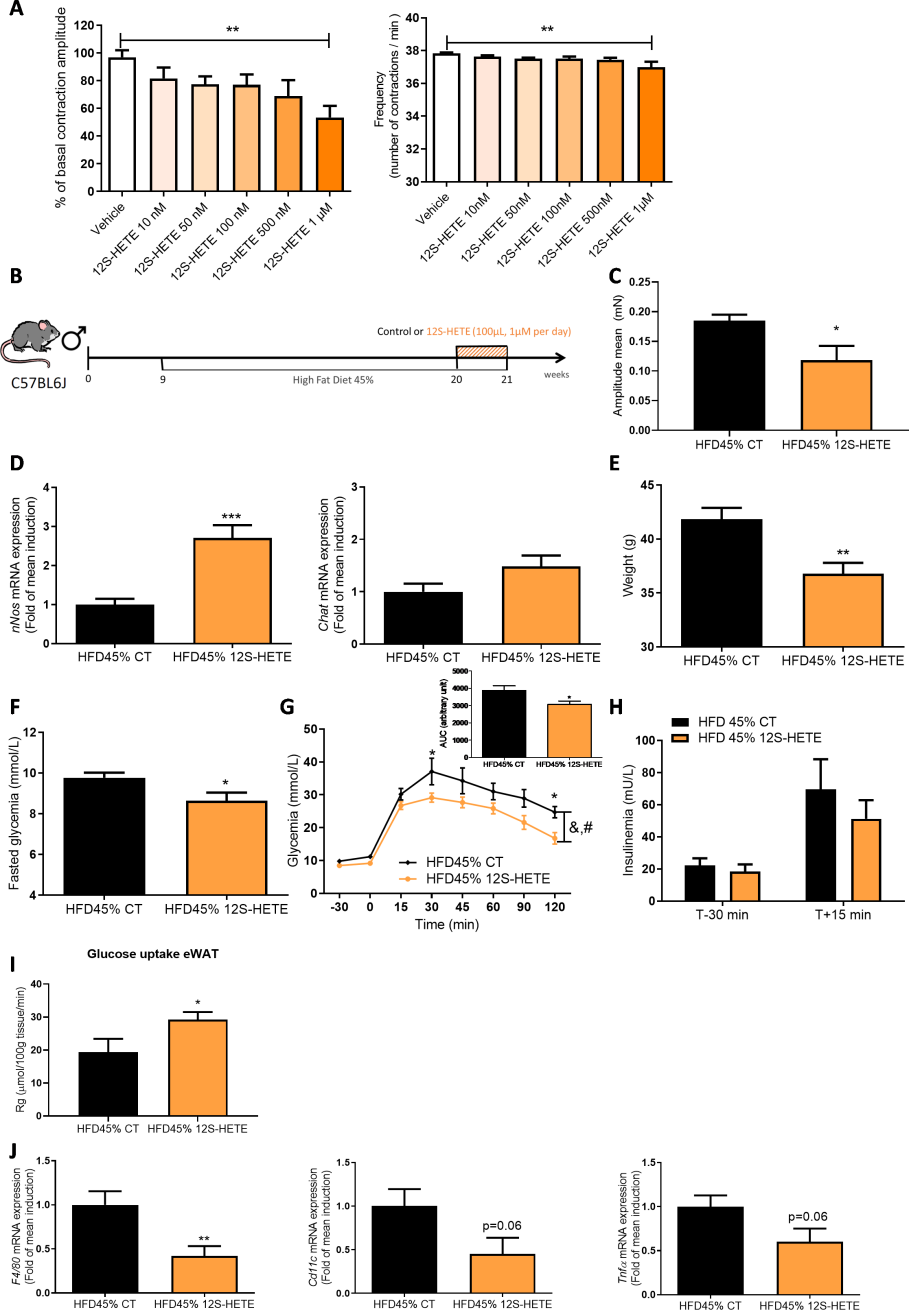
12S-HETE improves glucose metabolism and adipose tissue inflammation. (A) Ex vivo measurement of the duodenal mechanical contraction amplitude and frequency in response to Krebs-Ringer solution (vehicle) or 12S-HETE (from 10 nM to 1 µM, n=7). (B) Experiment designed to explore the metabolic effect of 12S-HETE on diabetic mice fed a HFD45%. (C) Ex vivo measurement of the duodenal mechanical contraction amplitude in chronic 12S-HETE treated mice (n=8–9 samples per group). (D) Expression of the *nNos* and *Chat* mRNAs in the duodenum (n=6 animals per group). (E) Body weight at the end of the experimental protocol (n=9–13 animals per group). (F) The results for fasting glycaemia. (G) Oral glucose tolerance test (OGTT) in mice fasted for 6 hour along with the area under the curve and (H) plasma insulin levels in mice treated with (n=13) or without (n=9) 12S-HETE are shown. (I) Glucose uptake (n=6–7 mice per group) and (J) expression of the *F4/80*, *Cd11c* and *Tnfα* mRNAs in the eWAT (n=8 animals per group). *p<0.05, **p<0.01 and ***p<0.001 compared with HFD45% CT; time effect: ^&^p<0001 and treatment effect ^#^p<0.05 (two-way ANOVA). ANOVA, analysis of variance; HFD, high-fat diet; 12S-HETE, 12S-hydroxyeicosatetraenoic acid.

We first aimed to evaluate the minimal efficient dose that potentially alters duodenal contraction. We first determined the most efficient dose of our compound that reduced intestinal contractions (figure 2A). We next used this dose to investigate whether 12-HETE contributed to improving the phenotype of obese and T2D mice fed a HFD45% (figure 2B).

Chronic oral treatment with 12S-HETE is associated with a specific decrease of duodenal amplitude of contraction (figure 2C) but not frequency (online supplemental figure S4B). However, 12S-HETE is the main metabolite of arachidonic acid in the brain and is produced by neurons to exert a neuroprotective effect by altering the transcription of PPARγ.^18^ Therefore, we investigated whether the effects of 12S-HETE were depending on PPARγ. We found that the decreased duodenal amplitude of contraction induced by 12S-HETE was blocked by the PPARγ antagonist GW9662 (online supplemental figure S4A, B).

The expression of the *nNos* mRNA, but not *Chat* mRNA, was significantly increased in the duodenum of mice that received a chronic oral treatment with 12S-HETE compared with the control group (figure 2D). This change was associated with decreases in body weight (figure 2E) and fasting blood glucose levels (figure 2F) and improved glucose tolerance (figure 2G), without changes in blood insulin levels (figure 2H). We investigated the effect of 12S-HETE on glucose metabolism by measuring tissue-specific glucose uptake using deoxy-D-glucose to understand the potential mechanism. According to our data, the improvement in glycaemia might be explained by a specific increase in glucose uptake in the eWAT of 12S-HETE-treated mice compared with control mice (figure 2I and online supplemental figure S4C–H). In addition, in mice fed a HFD45% supplemented with FOS, 12S-HETE supplementation decreased the expression of the *F4/80* mRNA compared with control HFD-fed mice (figure 2J; p=0.06 for *Cd11c* and *Tnfa* mRNA expression), suggesting that the eWAT appears to be the preferred target tissue of the effects of 12S-HETE on glucose homeostasis. Based on our data, 12S-HETE represents a potential molecular actor that targets the ENS-duodenal contraction couple to improve glucose homeostasis.

### PENK signalling is altered in the duodenum of patients with diabetes

The PPARγ agonist rosiglitazone activates the endogenous opioid PENK.^19^ In addition, 12S-HETE is also considered a second messenger of MOR,^15^ a receptor for enkephalin, and is able to inhibit neurotransmitter release.^15^

**Figure 3:**
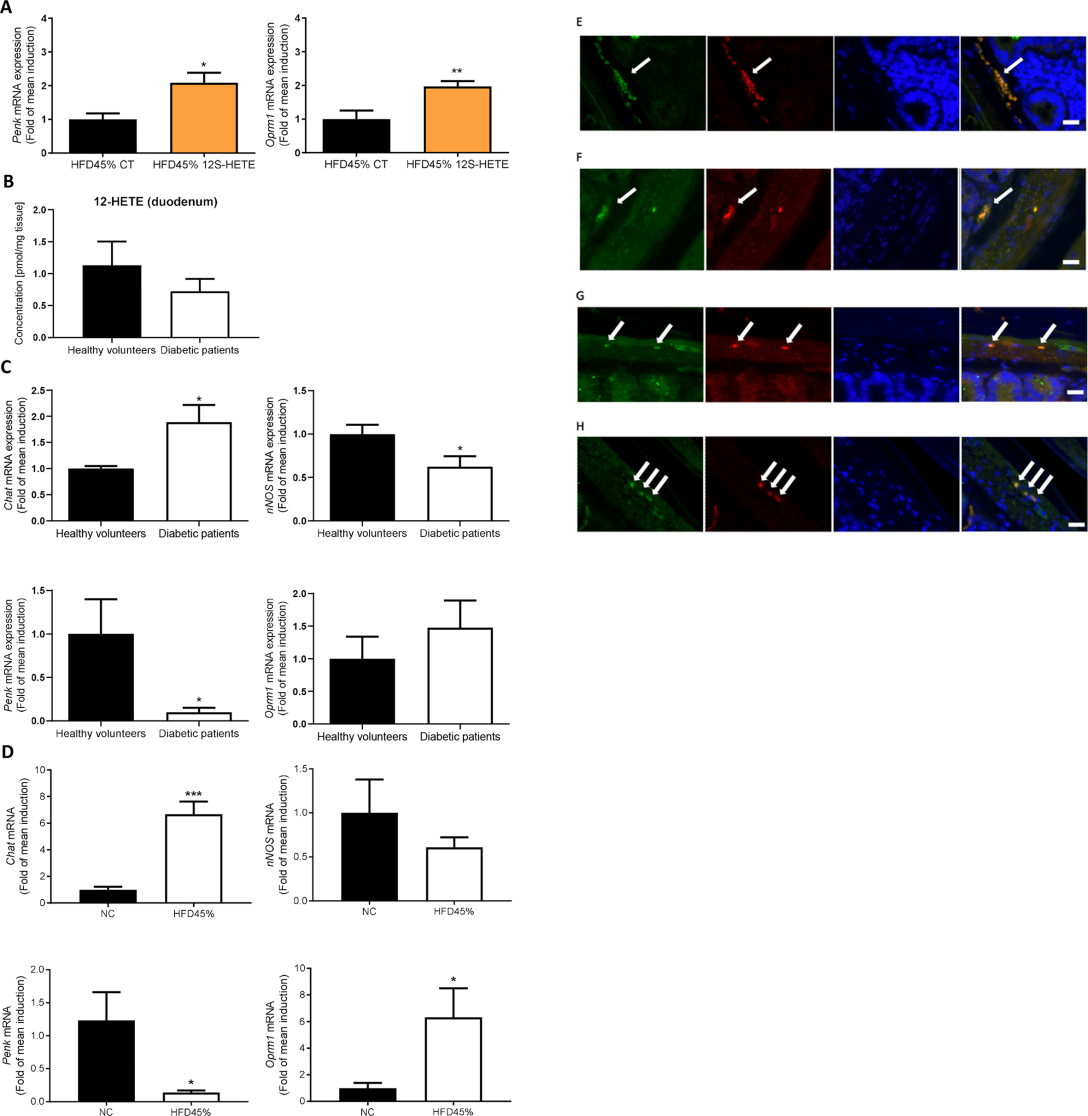
Proenkephalin signalling in humans and mice. (A) Expression of the *Penk* and *Oprm1* mRNAs in the duodenum of mice treated with or without 12S-HETE (n=6 mice per group). (B) Concentration of 12-HETE in the duodenum of healthy volunteers (n=5) or patients with diabetes (n=5). (C) Expression of the *Chat*, *nNos*, *Penk* and *Oprm1* mRNAs in the duodenum of healthy volunteers (n=5) or patients with diabetes (n=6). (D) Expression of the *Chat*, *nNos*, *Penk* and *Oprm1* mRNAs in the duodenum of control mice or mice fed a HFD45% (n=5 mice per group). (E) Immunochemical staining of ChAT-expressing neurons (green) and MOR (red) in the ENS. (F) Immunochemical staining of nNOS-expressing neurons (green) and MOR (red) in the ENS. (G) Immunochemical staining of ChAT-expressing neurons (red) and Alox12 (green) in the ENS. (H) Immunochemical staining of nNOS-expressing neurons (red) and Alox12 (green) in the ENS. White arrows indicate the enteric neurons and the colocalisation. Bars=10 µm. ENS, enteric nervous system; HFD, high-fat diet; MOR, mu-opioid receptors; 12S-HETE,12S-hydroxyeicosatetraenoic acid.

We have first determined the link between the PENK signalling pathway and 12S-HETE by measuring the expression of PENK and its receptors in 12S-HETE-treated mice. Notably, 12S-HETE increased the expression of the *Penk* and *Oprm1* mRNAs (figure 3A).

We compared the expression profiles of these markers and measured the quantity of 12-HETE in duodenal biopsies obtained from healthy volunteers and patients with T2D to further explore whether the data obtained from rodents might translated to humans. We first observed a 38% decrease of 12-HETE concentration in the duodenum of patients with diabetic but did not reach significance (figure 3B). We observed an increase in the expression of the *Chat* mRNA, a decrease in the expression of the *nNos* and *Penk* mRNAs, and no significant effect on *Oprm1* expression in the duodenum of subjects with T2D (figure 3C) compared with healthy volunteers. Interestingly, similar results were observed in the duodenum of diabetic mice, such as an increase in the duodenal expression of the *Chat* mRNA and a decrease in the expression of the *Penk* mRNA, with no effect on *nNos* expression (figure 3D). In contrast to humans, the expression of the *Oprm1* mRNA was markedly increased in the duodenum of diabetic mice (figure 3D), suggesting a compensatory effect of the altered PENK signalling. We next localised MOR receptors in mice using immunohistochemistry. These receptors were expressed on ChAT-expressing (figure 3E) and nNOS-expressing (figure 3F) neurons in the ENS. In addition, the presence of the 12-HETE-synthetising enzyme Alox12 was observed in ChAT-expressing (figure 3G) and nNOS-expressing (figure 3H) neurons in the ENS.

### MOR controls duodenal contractions and glucose metabolism

**Figure 4:**
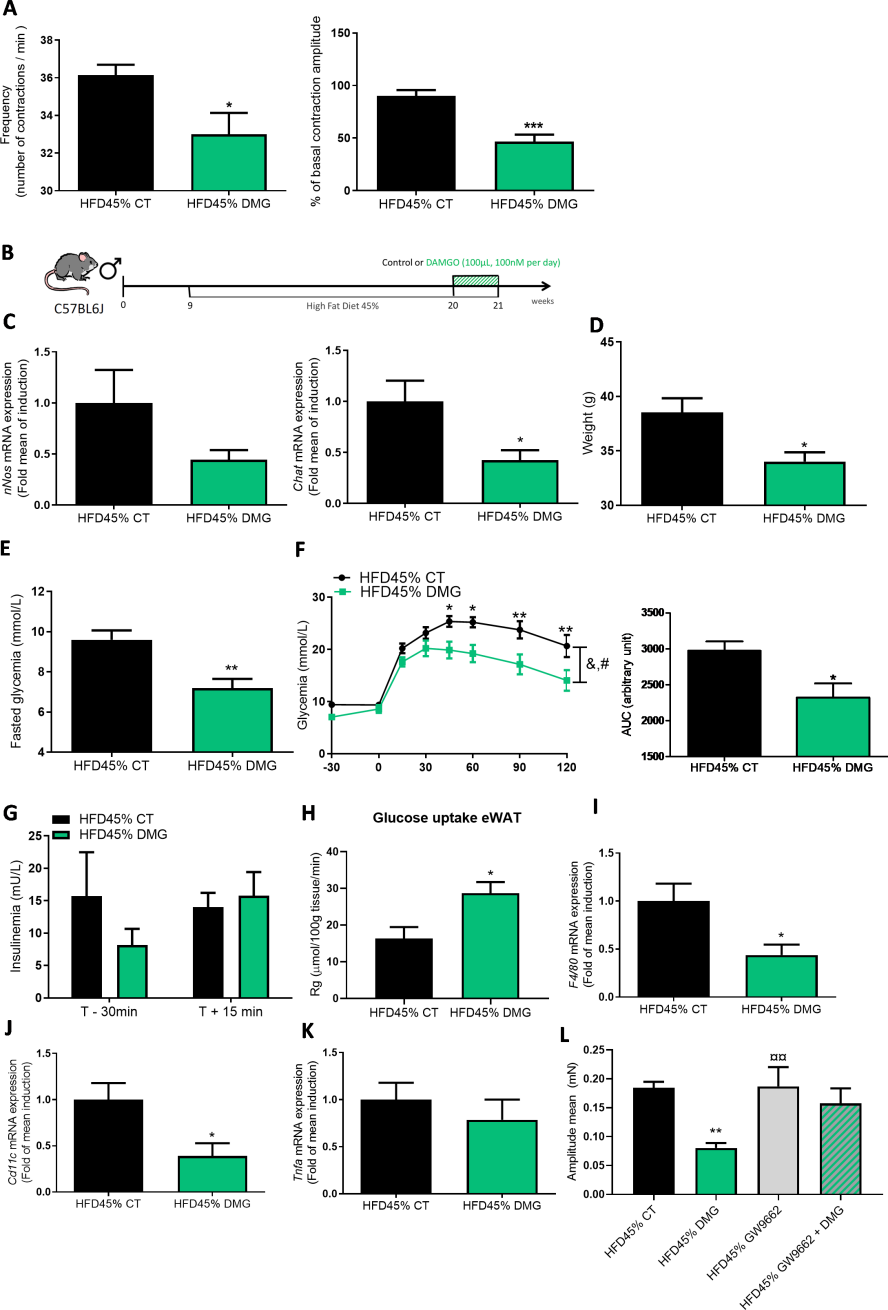
DAMGO improves glucose metabolism and adipose tissue inflammation. (A) Ex vivo measurement of the duodenal mechanical contraction amplitude and frequency in response to Krebs-Ringer solution (Vehicle) or DAMGO (100 nM) (n=7 per group). (B) Experiment designed to describe the metabolic effect of DAMGO on diabetic mice fed a HFD45%. (C) Expression of the *nNos* and *Chat* (n=4–5 mice per group) mRNAs in the duodenum. (D) Body weight at the end of the experimental protocol (n=8 mice per group). (E) Fasting glycaemia. (F) Oral glucose tolerance test (OGTT) in mice fasted for 6 hour along with the area under the curve. (G) Plasma insulin levels in mice treated with or without DAMGO (n=7–12 per group). (H) Glucose uptake (n=5–6 mice per group) (I) Expression of the *F4/80*, J. *Cd11c* and K. *Tnfα* mRNAs in the eWAT (n=7–10 per group). (L) Ex vivo measurement of the duodenal mechanical contraction amplitude in chronic DAMGO treated mice (n=7–9 samples per group). *p<0.05, **p<0.01,***p<0.001 and ****p<0.0001 compared with HFD45% CT; time effect: &p<0.0001 and treatment effect #p<0.0001 (two-way ANOVA). **p<0.01 versus HFD45% CT, ¤¤p<0.01 versus HFD45% DMG (one-way ANOVA). ANOVA, analysis of variance; Chat, choline acetyl transferase; eWAT, epididymal white adipose tissue; HFD, high-fat diet.

To determine whether MOR is a receptor controlling duodenal contractions and glucose metabolism, we used the MOR agonist ‘DAMGO’.^20^ Consistent with the observations from mice treated with 12S-HETE, the MOR agonist decreased the mechanical activity of the duodenum of diabetic mice (figure 4A). To further investigate the potential effect of the MOR agonist in vivo, we chronically administered DAMGO by oral gavage (figure 4B). We observed a decrease in the expression of the *Chat* mRNA, but not *nNos* mRNA, in the duodenum of DAMGO-treated mice (figure 4C). Diabetic mice treated with DAMGO presented a significant reduction in body weight (figure 4D), a decrease in fasting blood glucose levels (figure 4E) and improved glucose tolerance (figure 4F), without modifications in insulin release (figure 4G). The DAMGO treatment increased glucose entry in the eWAT (figure 4H), but not in other tissues (online supplemental figure S6A-F), similar to the findings obtained with 12S-HETE. Again, the expression of proinflammatory markers in eWAT was decreased in DAMGO-treated mice (figure 4I–K). In addition, we observed an increase in the expression of the *Ucp1* mRNA in eWAT, but not other potential markers of mitochondrial biogenesis, such as *Pgc1a* and *Prdm16* (online supplemental figure S6G). This treatment is also associated with a decrease of the mean amplitude of contraction in the duodenum (figure 4L) but not frequency (online supplemental figure S5). To further explore whether the effects of DAMGO were depending on PPARγ, we used the PPARγ antagonist and found that the mean amplitude of contraction was also blocked by GW9662, thereby suggesting that the second messenger 12S-HETE contributed to DAMGO effects.

## Discussion

The importance of ENS in controlling glucose metabolism is becoming clear.^7^ We identified two novel enterosynes (12S-HETE and enkephalin) that are modulated by the gut microbiota as well as novel mechanisms of action and metabolic effects of the bioactive lipid 12S-HETE. The identification of novel tools to treat T2D is urgently needed since the large majority of antidiabetics have been associated with either side effects (nausea, diarrhoea etc) or difficulty in administration (ie, injection). As shown in our previous study, pharmacological approaches using apelin^5^ and galanin^6^ modulate the activity of enteric neurons to control glycaemia. We have used a nutritional approach employing the prebiotic FOS to identify novel enterosynes. The beneficial effect of FOS on glucose metabolism has been extensively described in the literature,^21^ and its actions require the activities of numerous biological factors controlled by gut microbiota that include SCFA, neurotransmitters and hormones.^8^ However, the effect of prebiotics on the production of different bioactive lipids is poorly studied. Therefore, we screened different bioactive lipids and found that the prebiotic-induced modulation of the gut microbiota increased the level of 12-HETE in the colon, a bioactive lipid derived from arachidonic acid. Studies aiming to determine the role of bioactive lipids in the pathophysiology of T2D are in their infancy, and a particular focus is the interaction between lipids and inflammation.^22^ Indeed, 12S-HETE signalling is implicated in the control of multiple physiological functions, and it could be associated with deleterious^23^ or beneficial^18^ effects, including neuroprotection, as in the case of MOR^24^ and PPARγ signalling.^25^ In our experimental model, only the level of 12-HETE was significantly increased in the colon of mice fed a HFD45% supplemented with FOS. The pharmacology of 12S-HETE is relatively complex and depends on the route of administration.^23^ Some of its effects are associated with a pancreatic proinflammatory action that participates in the establishment of T2D. In contrast, T2D is characterised by an alteration in the ENS and the loss of specific neurons.^26^ In the pathological state, neurons are able to release 12S-HETE and generate an endogenous neuroprotective signal.^18^ In this case, 12S-HETE may be considered a protective signal in the gut for enteric neurons. This hypothesis is reinforced by the fact that 12S-HETE modifies the intestinal expression of *Penk*. In addition, enkephalin exerted an antiinflammatory action in the gut in our previous study.^27^ The global effects of a chronic oral 12S-HETE treatment on glycaemia were positive in our experimental model, suggesting that its action is limited to the gut. In addition, we discovered that mechanistically 12S-HETE requires the activation of PPARγ to reduce duodenal contractility.

As 12S-HETE is also the second messenger of the enkephalin/MOR pathway, we must focus on this pathway to confirm that future therapeutic approaches will not exert side effects. Here, by using an MOR agonist, we have observed a decrease in body weight and no change in insulin release in response to oral administration, revealing an improvement in insulin sensitivity. Accordingly, in a recent study, intraperitoneal injections of enkephalin in streptozotocin-induced diabetic rats decrease glycaemia.^28^ The authors also showed that enkephalin preferentially acts by targeting the pancreas to stimulate insulin release. Taken together, these two studies tend to show that strategies modulating the ENS are innovative due to their ‘local’ mode of action, thereby limiting potential side effects in peripheral tissues. In addition to the facilitation of the medication, the oral therapeutic approach described in our study suggests that it may be a useful treatment for patients with diabetes who also suffer from obesity.

In the human duodenum, we found a 38% decrease of 12-HETE concentration in patients with diabetic compared with healthy volunteers, but this effect was not statistically significant. This could be due to numerous experimental factors that could include the limited number of patients, antidiabetic drug treatment taken before sample collection and/or potential variation of time for biopsies after death rendering difficult the biological assay of bioactive lipids.

As shown in our previous study, the modulation of ENS activity is associated with changes in glucose entry in different tissues. Chronic oral apelin administration^5^ modulates glucose entry in muscles and oral galanin administration^6^ alters glucose entry in the liver, muscles and adipose tissue by acting on nNOS-expressing and/or ChAT-expressing neurons. Here, we have discovered that FOS, 12S-HETE and enkephalin exert effects on enteric neurons that correlate with an anti-inflammatory effect on adipose tissue. These results are consistent with previous studies showing that an FOS treatment decreases body weight and particularly adipose tissue weight and changes that are associated with decreased expression of proinflammatory markers.^29^ The modulation of the couple ‘enteric neurons-duodenal contraction’ impacts the brain,^5 6^ but other mechanisms of action are expected and may exert complementary effects.

**Figure 5:**
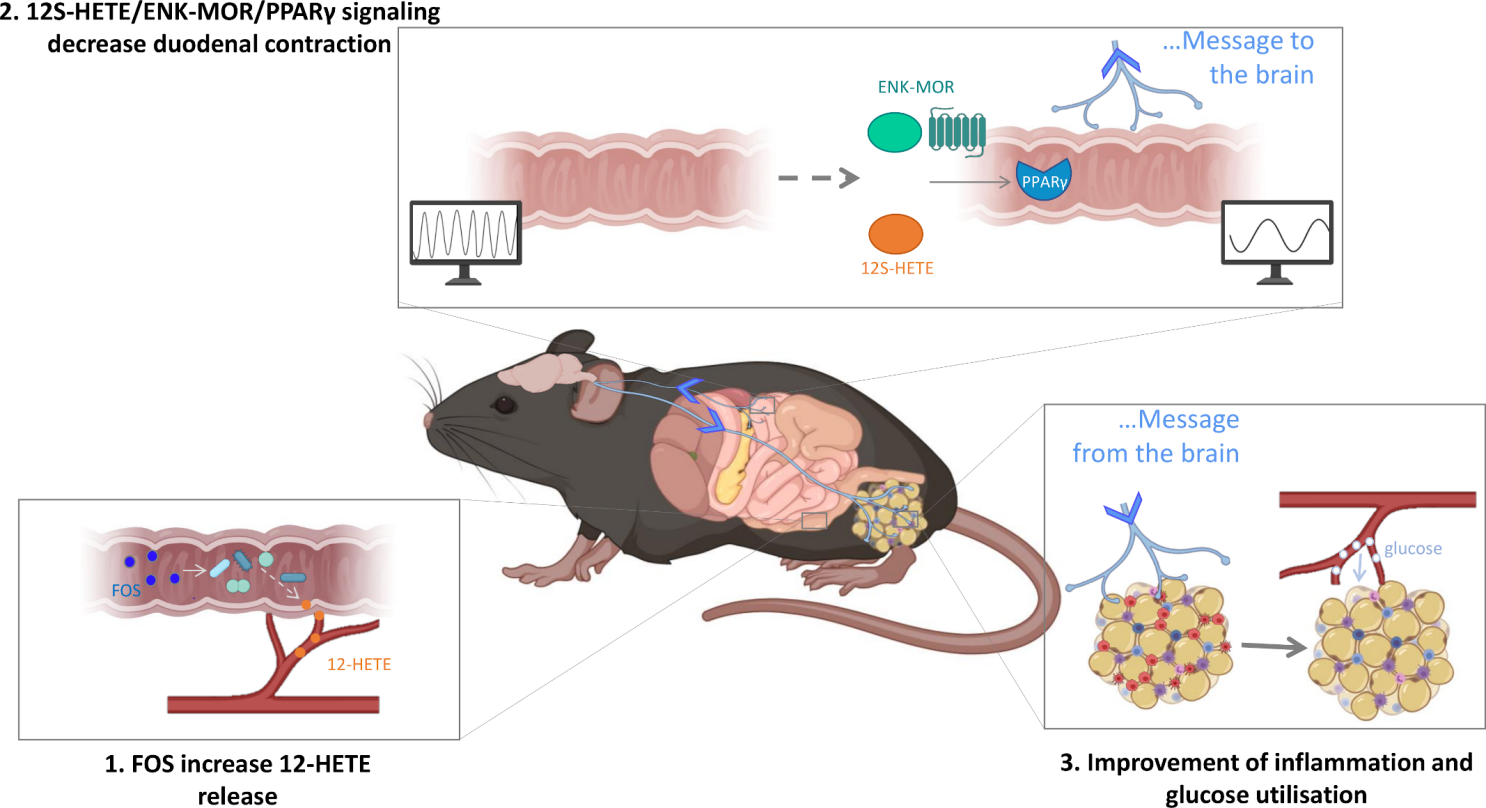
Recapitulative scheme. We identify that the modulation of the gut microbiota with FOS supplementation increases 12-HETE release in the colon of mice (point 1). This is associated with a decrease of duodenal contraction through 12S-HETE/ENK-MOR/PPARγ signalling (point 2). The modulation of ENS/contraction couple by these new enterosynes leads to an improvement of the inflammatory state and glucose utilisation in the white adipose tissue of diabetic mice (point 3). ENK, enkephalin; FOS, fructooligosaccharides; HFD, high-fat diet; PPARγ, peroxisome proliferator-activated receptor gamma.

In conclusion, our work provides new elements that improve our understanding of the mode of action of novel enterosynes in the control of glycaemia that control duodenum contraction *via* a PPARγ signalling (figure 5). Using a combination of nutritional and pharmacological approaches, we have identified a new mode of communication between gut microbes and the host. In addition, we have identified novel targets and their mechanisms of action in rodents and possibly in humans. The identification of specific targets, such as the enteric neuronal population, to treat T2D and its comorbidities represent a ground-breaking solution to develop medications without side effects.

## Data Availability

Data are available upon reasonable request. Data are available upon reasonable request to claude.knauf@inserm.fr.
