# Supplementary figures and images for: Identification of new enterosynes using prebiotics: roles of bioactive lipids and mu-opioid receptor signalling in humans and mice
Source: Gut. 2020 Oct 5;70(6):1078–87. doi: 10.1136/gutjnl-2019-320230 (PMC8108281; doi:10.1136/gutjnl-2019-320230)

Suppl. Figure 1

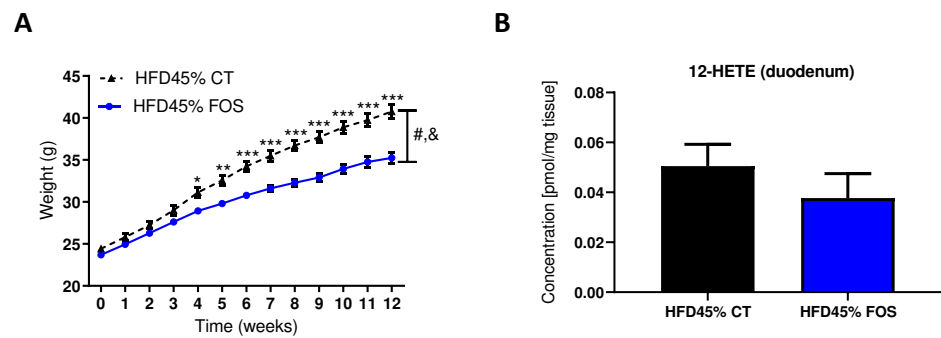

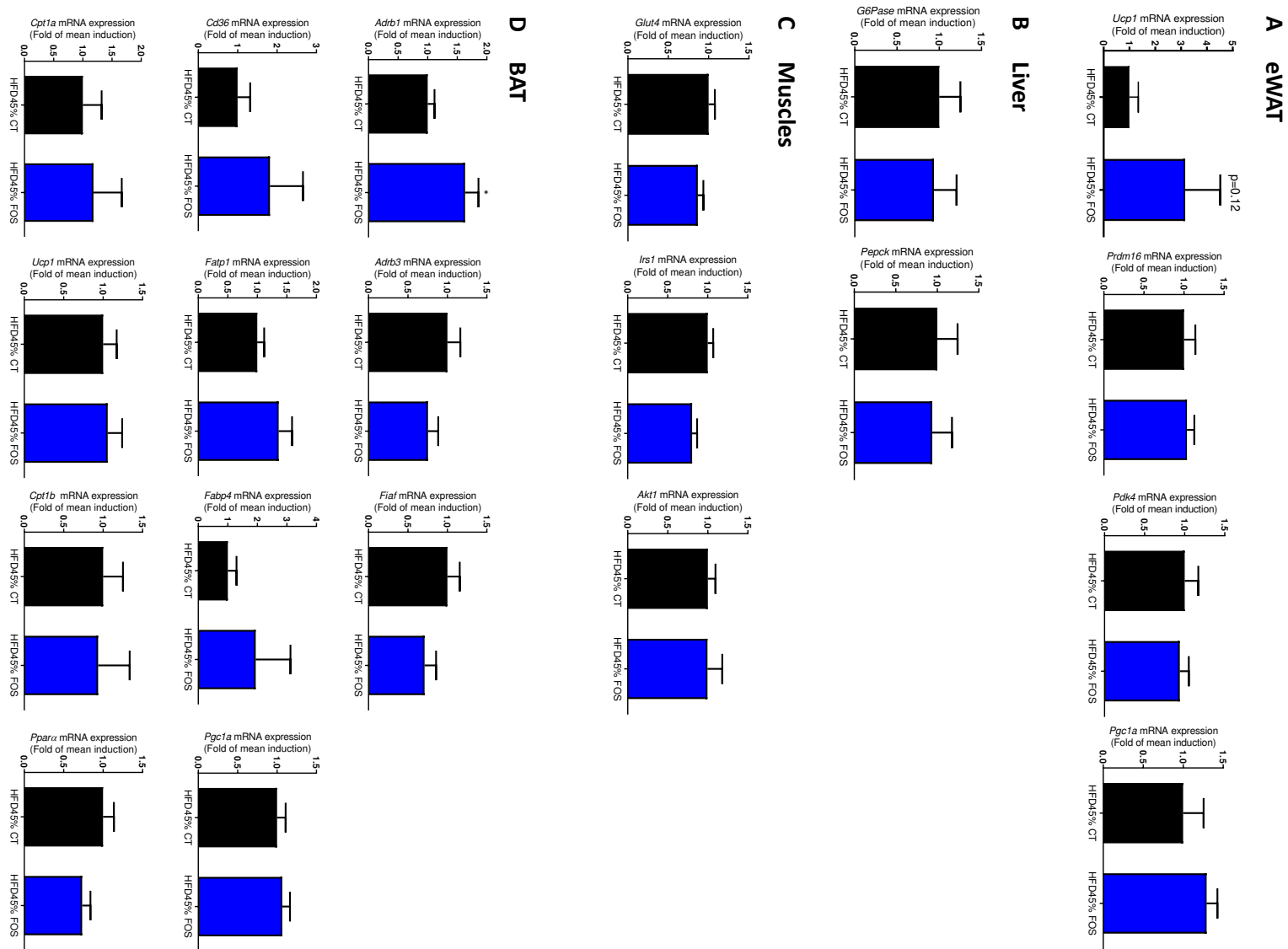

Suppl. Figure 2

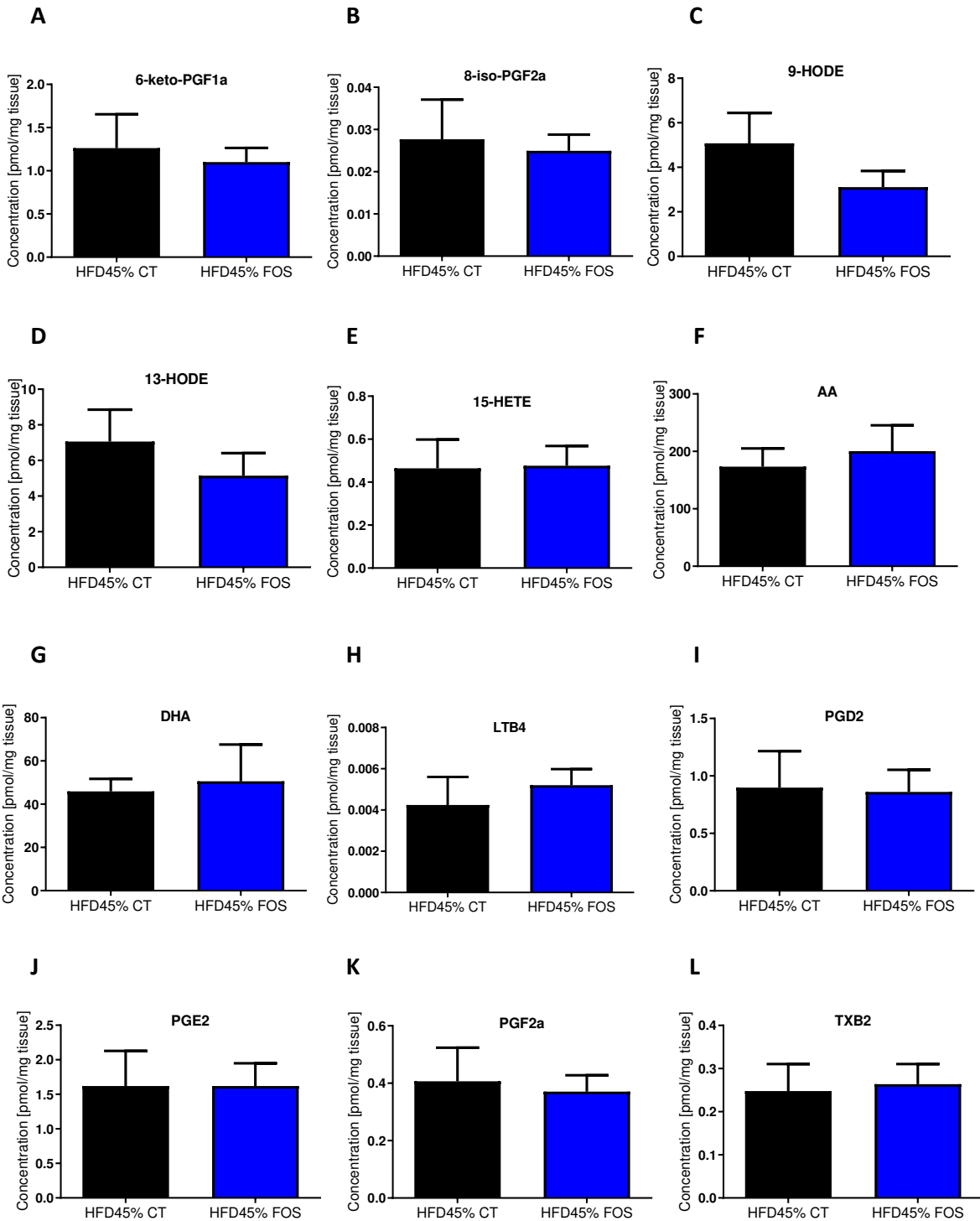

Suppl. Figure 3

Suppl. Figure 4

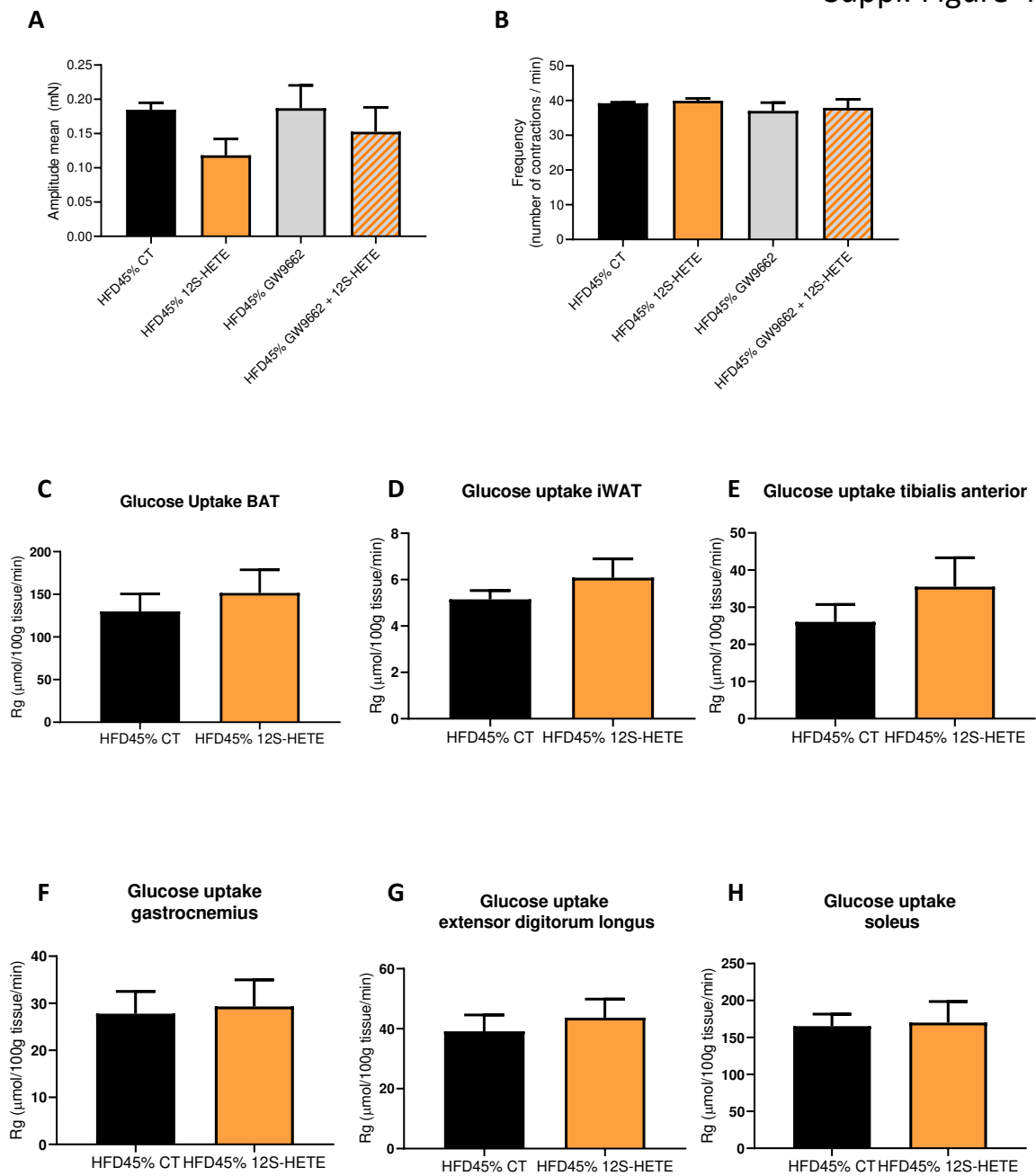

Suppl. Figure 5

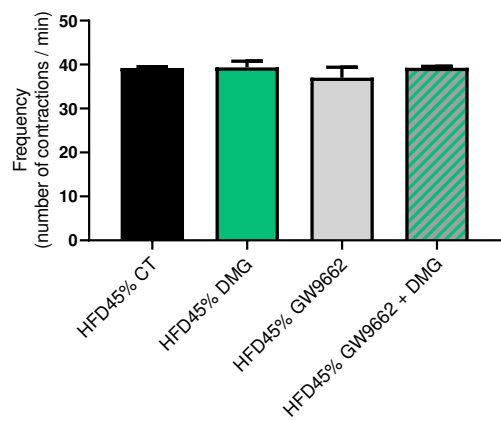

Suppl. Figure 6

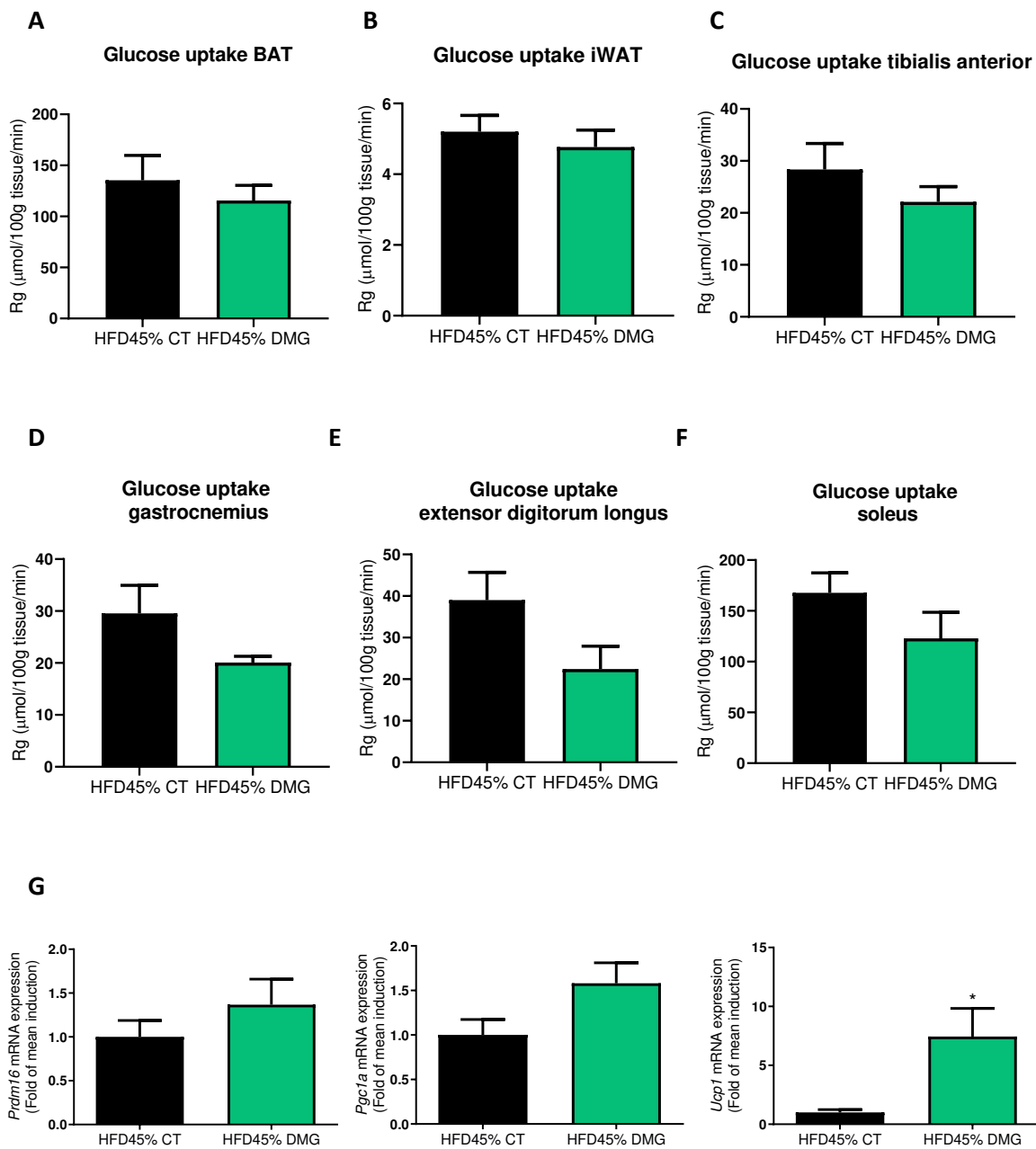

Supplement: Supplementary data [file gutjnl-2019-320230supp001.pdf]
